# Dose-Dependent Regulation on Prefrontal Neuronal Working Memory by Dopamine D_1_ Agonists: Evidence of Receptor Functional Selectivity-Related Mechanisms

**DOI:** 10.3389/fnins.2022.898051

**Published:** 2022-06-16

**Authors:** Yang Yang, Susan D. Kocher, Mechelle M. Lewis, Richard B. Mailman

**Affiliations:** ^1^Department of Pharmacology, Penn State Milton S. Hershey Medical Center, Penn State College of Medicine, Hershey, PA, United States; ^2^Translational Brain Research Center, Penn State Milton S. Hershey Medical Center, Penn State College of Medicine, Hershey, PA, United States; ^3^Department of Neurology, Penn State Milton S. Hershey Medical Center, Penn State College of Medicine, Hershey, PA, United States

**Keywords:** dopamine D1 agonist, dose response analysis, prefrontal cortex, working memory, functional selectivity/biased agonism

## Abstract

Low doses of dopamine D_1_ agonists improve working memory-related behavior, but high doses eliminate the improvement, thus yielding an ‘inverted-U’ dose-response curve. This dose-dependency also occurs at the single neuron level in the prefrontal cortex where the cellular basis of working memory is represented. Because signaling mechanisms are unclear, we examined this process at the neuron population level. Two D_1_ agonists (2-methyldihydrexidine and CY208,243) having different signaling bias were tested in rats performing a spatial working memory-related T-maze task. 2-Methyldihydrexidine is slightly bias toward D_1_-mediated β-arrestin-related signaling as it is a full agonist at adenylate cyclase and a super-agonist at β-arrestin recruitment, whereas CY208,243 is slightly bias toward D_1_-mediated cAMP signaling as it has relatively high intrinsic activity at adenylate cyclase, but is a partial agonist at β-arrestin recruitment. Both compounds had the expected inverted U dose-dependency in modulating prefrontal neuronal activities, albeit with important differences. Although CY208,243 was superior in improving the strength of neuronal outcome sensitivity to the working memory-related choice behavior in the T-maze, 2-methyldihydrexidine better reduced neuron-to-neuron variation. Interestingly, at the neuron population level, both drugs affected the percentage, uniformity, and ensemble strength of neuronal sensitivity in a complicated dose-dependent fashion, but the overall effect suggested higher efficiency and potency of 2-methyldihydrexidine compared to CY208,243. The differences between 2-methyldihydrexidine and CY208,243 may be related to their specific D_1_ signaling. These results suggest that D_1_-related dose-dependent regulation of working memory can be modified differentially by functionally selective ligands, theoretically increasing the balance between desired and undesired effects.

## Introduction

The prefrontal cortex (PFC) subserves higher-order cognitive function, and its neuron activities represent the cellular basis of working memory (WM) (Goldman-Rakic, 1995, 2011; Kesner and Churchwell, 2011; Caetano et al., 2012; Laubach et al., 2015; Yang and Mailman, 2018). Dopamine D_1_ receptors (D_1_Rs) play important roles in the PFC (Murphy et al., 1996a; Lidow et al., 2003; Arnsten et al., 2015), and D_1_ agonists cause marked cognitive improvement in laboratory animals (Arnsten et al., 1994, 2017; Murphy et al., 1996b; Cai and Arnsten, 1997; Zahrt et al., 1997; Vijayraghavan et al., 2007; Wang et al., 2019; Yang et al., 2021) and in humans (Mu et al., 2007; Rosell et al., 2015; Huang et al., 2020). In animal studies where dose can be manipulated, low doses of dopamine D_1_ agonists improve working memory-related behavior, but high doses eliminate the improvement, thus yielding an ‘inverted-U’ dose-response curve (Arnsten et al., 1994; Cai and Arnsten, 1997; Zahrt et al., 1997; Vijayraghavan et al., 2007; Yang et al., 2021). In monkeys performing spatial WM (sWM) tasks, low doses of D_1_ agonists enhanced spatial tuning of a single PFC neuron by increasing responses to preferred directions or suppressing responses to non-preferred directions. Conversely, high doses changed the firing for all directions, eroding tuning (Vijayraghavan et al., 2007; Wang et al., 2019). These dose-dependent sculpting actions at the single neuron level could be the cellular basis of the inverted-U dose response of D_1_ agonists at the behavioral level. Alternately, neural ensembles in the PFC may be more important than single neurons (Jung et al., 1998; Baeg et al., 2003; Horst and Laubach, 2012; Yang et al., 2014; Bolkan et al., 2017; Murray et al., 2017; Spaak et al., 2017; Yang and Mailman, 2018; De Falco et al., 2019). This makes the dose-dependent analysis of D_1_ action at the neuron population level of special importance at both the basic and translational levels.

The pattern of signal transduction mediated by a drug acting at single receptor (commonly called *functional selectivity* or *biased signaling*) is recognized to be of heuristic importance, but also offers the possibility of developing novel therapies (Urban et al., 2007; Kenakin, 2012). Cyclic AMP (cAMP) is the canonical intracellular messenger mediated by D_1_Rs, and a key player in dose-dependent regulation at the single neuron level in the PFC (Vijayraghavan et al., 2007). G protein-independent β-arrestin-related signaling also may be critical (Urs et al., 2011, 2015; Liu et al., 2015; Yang et al., 2021). β-Arrestin, besides functions for receptor desensitization and internalization, also acts as a multifunctional signal transducer by serving as an adaptor/scaffold to connect the activated receptors with diverse signaling pathways within the cell (Yang, 2021). We hypothesize that both cAMP and β-arrestin are involved in the dose-dependent regulation of D_1_ agonists, as are other D_1_R-mediated signaling such as opening/closing of ion channels (Paspalas et al., 2013; Arnsten, 2015; Arnsten et al., 2015; Gamo et al., 2015). Our underlying premise was that functionally selective/biased D_1_ agonists differ in their dose-response characteristics based on differential engagement of alternate signaling pathways. We used two D_1_ selective agonists (2-methyldihydrexidine and CY208,243) that contrast in their signaling bias (Yang et al., 2021) to compare dose-response effects in a rodent sWM task. 2-Methyldihydrexidine has modest bias toward D_1_-mediated β-arrestin-related signaling as it is a full agonist at adenylate cyclase and a super-agonist (activity greater than dopamine) at β-arrestin recruitment, whereas CY208,243 is slightly bias toward D_1_-mediated cAMP signaling with relatively high intrinsic activity at adenylate cyclase, but only partial agonism at β-arrestin recruitment. We probed three aspects of neuron population dynamics in the PFC: percentage; uniformity; and ensemble strength of neuronal sensitivity (see Methods). The results indicate that D_1_ agonists affect neuron population dynamics in the PFC in a complicated dose-dependent manner. These data also suggest that functional selectivity can be a promising strategy for the discovery of novel D_1_ ligands that may have an improved therapeutic index for cognition.

## Materials and Methods

### Subjects

All animal care and surgical procedures were in accordance with the National Institutes of Health Guide for the Care and Use of Laboratory Animals and Penn State Hershey Animal Resources Program, and were reviewed and approved by the local IACUC. A total of six male Sprague-Dawley rats (Charles River Laboratories) weighing 226–350 g when received were housed individually and maintained on a 12-h light-dark cycle with water continuously available. They were fed a limited diet of Bio-Serv rat chow (5 g/100 g) to maintain their body weight at 90–95% of free-feeding body weight for motivation purposes. Highly palatable rewards (chocolate flavored sucrose, Bio-Serv) were used during testing.

### Drug Preparation and Administration

2-Methyldihydrexidine (2MDHX) was synthesized by modifications of published procedures (Yang et al., 2021) whereas CY208,243 (CY208) was purchased from Tocris (Minneapolis, MN, United States). Both compounds were of >97% purity. Stock solutions of 100 mM 2MDHX and CY208 were made in DMSO and stored at −80°C in the dark. For use, they were diluted in 0.1% ascorbic acid vehicle using a dose range (1, 10, 100, and 10,000 nmol/kg) suggested by prior experiments (Yang et al., 2021). Working solutions were prepared on the day of experiments, and injected subcutaneously under brief ca. 4% isoflurane anesthesia. Rats recovered within a minute, and then were placed in the test arena to habituate to the environment. The behavioral task and electrophysiological recording started ca. 20 min later. The order of drug condition was randomly assigned and the interval between two drug conditions was at least 5 days.

### Microwire Electrode Array Implantation in Medial Prefrontal Cortex

Rats were given 1 week of full access to food before unilateral implantation of a microwire electrode into the right medial PFC (mPFC) (Yang and Mailman, 2018). Animal weights were all over 350 g when craniotomy surgeries were performed. After initial anesthesia with ca. 4% isoflurane, a continuous 0.5–2% isoflurane anesthesia was maintained during the surgery. The animal was fixed on a stereotactic frame and ophthalmic antibiotic ointment was applied to prevent the eyes from desiccation. The incised site was disinfected and subcutaneously injected by drops of bupivacaine. The skull surface was exposed and adjusted to lie flat between Bregma and Lambda. Four small holes were drilled for anchor screws including one that served as a ground for the electrode. A 2.5 mm × 2.5 mm bone window was made above the mPFC and the dura mater carefully was removed. A microwire electrode array targeting the mPFC was lowered vertically to a depth of 3.5 mm from the brain surface at 3.0 mm rostral to bregma and 0.4 mm lateral to bregma. The microwire electrode array was made with 25 μm stainless steel wire coated by polyimide (H-ML), which has an impedance between 600 and 900 kΩ, arranged in a 2 × 4 configuration with 250 μm between electrodes (MicroProbes). The array was positioned with its long axis parallel to the anterior-posterior plane. After electrode placement, the craniotomy was sealed with dental cement and wound margins were daubed with antibiotic ointment. Rats were given enrofloxacin (5 mg/kg, Baytril, Bio-Serv) and carprofen (20 mg/kg, Rimadyl, Bio-Serv) tablets for 3 days, and had full access to food for at least 1 week.

### Delayed Alternation Response Task in the T-Maze

**FIGURE 1:**
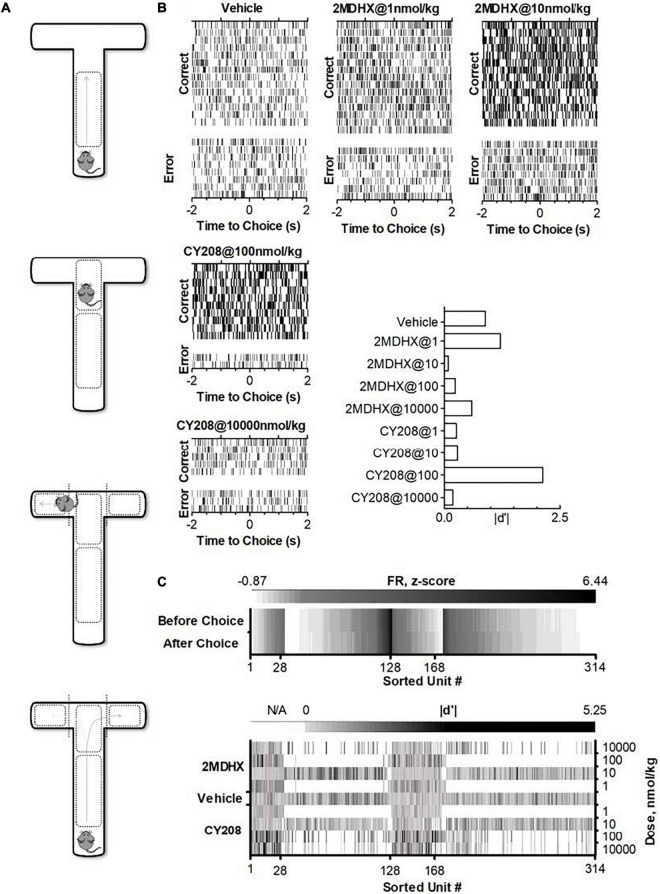
Experimental paradigm and single neuron activities after administration of D_1_ agonists 2MDHX or CY208. **(A)** Standard T-maze. Pre-defined zones and grids were used to indicate an animal’s behavior as making a choice (passed grids). **(B)** A single neuron example shows the dose response after 2MDHX or CY208 administration. Rasters indicate individual spikes. Trials are organized by correct vs. error outcome. For better illustration, only example trials for five drug conditions were shown, but this neuron was tested for all eight drug conditions. The effect on outcome sensitivity by different doses of drug was summarized in the histogram at the lower-right corner. Note CY208 (at 100 nmol/kg) increased its sensitivity strength better than 2MDHX (at 1 nmol/kg), but its “optimal” and “detrimental” doses of 2MDHX (1 and 10 nmol/kg, respectively) were both lower than CY208 (100 and 10,000 nmol/kg, respectively). **(C)** Summary of all recorded single neurons. Top panel shows firing rate (FR, z-score) during control conditions where vehicle was administered. Note that units #1–128 are prospective-encoding-neurons that have a higher FR before the WM-related choice behavior during the DAR task (i.e., top row has darker color). Conversely, units #129–314 are retrospective-encoding-neurons that have a higher FR after the choice (i.e., bottom row has darker color). The bottom panel shows the strength of outcome sensitivity (*|d′|*) during each drug condition (2MDHX/CY208 @ 1/10/100/10,000 nmol/kg). N/A means data not available as this drug condition was not tested. Only 28 prospective-encoding-neurons (units #1–28) and 40 retrospective-encoding-neurons (units #129–168) were tested for all eight drug conditions.

Rats individually were trained in a T-maze and acclimated with the electrophysiological recording setup (Yang and Mailman, 2018). A standard T-maze was utilized that had one start runway (56 cm long, 10 cm wide and 18 cm high) and two finish arms (41 cm long, 10 cm wide and 18 cm high). The maze was made of acrylic polycarbonate with a black floor and clear sides for better video and electrophysiological recordings. A CCD camera (30 frames/second, STC-TB33USB-AS, SenTech) was hung over the top of the maze and connected to a Limelight video recording system (Actimetrics, Coulbourn Instruments) to monitor simultaneously the animal’s free movement in the maze. Pre-defined grids (Figure 1A) indicated the animal’s behavior (passed grids). The Limelight system sent out a TTL (+5V) signal to mark the reference time (RT), referring to the time for choice behavior (passed a grid).

Rats were habituated to all procedures and tested by a single person. To administer drugs and connect the electrophysiological recording cables, rats were anesthetized briefly with ca. 4% isoflurane. They recovered within a minute and were placed in the recording arena to habituate to the environment. The delayed alternation response (DAR) task started after ca. 20 min and began when the animal was placed in the start box of the T-maze. The start box is located in the lower section of the start runway and cordoned off by a solid gate. After the tester raised the gate, the rat needed to run to the intersection of the T and make a choice of turning down the left or right arm of the maze. This first choice always was rewarded with a hand-fed food reward at the end of the finish arm. Then the rat gently was returned to the start box and remained there for a predetermined fixed delay (5 s), as this is the general temporal scale of WM tasks. After the delay elapsed, the gate was raised, and the task was repeated. For each trial, rats needed to run to the intersection, make a choice, and reach the end of the arm in less than 1 min, otherwise the trial was aborted, and the DAR task restarted. This continued for ca. 40 min to be considered a complete test session. The rats were trained to visit the two arms of the maze alternatively, and were rewarded only after a correct choice. During the entire procedure, the tester minimized any possible cues that might affect choice.

All rats were well-trained for the task. They made correct choice >60% during the vehicle control condition after which they underwent multiple test sessions to evaluate drug effects. Each session had ca. 20 trials (range 8–97). There was a minimum of 5 days of drug washout between any two drug test sessions.

### Electrophysiological Recordings

Neural recordings were collected during DAR task using the OmniPlex Neural Data Acquisition System (Plexon) that also recorded the RT signal from Limelight, which enable synchronization of neural and behavioral recordings. Wideband and spike signals were recorded for later off-line analysis. The wideband signal was digitized at 40 kHz. A highpass filter with a cutoff of 250 Hz yielded the continuous spike signal that was sampled at the same 40 kHz rate as the original wideband signal. Artifacts due to cable noise and devices were removed during off-line analysis.

Action potentials were detected and sorted both on- and off-line *via* the Offline Sorter (Plexon) to get better unit isolation results. Waveforms from multiple units were sorted by means of voltage-time threshold windows and a two principal components (PCs) contour template algorithm (PCA). The degree to which the waveform clusters are separated in the 2D projection of two PCs was determined by a Multivariate ANOVA test. Significance (α < 0.05) indicated that each waveform cluster had a different location in 2D space and that the clusters were well separated. Then, each well-separated waveform cluster was assigned as a single unit. The same sorting method was implemented throughout all recording sessions, ensuring the sorting stability of the waveform of a single neuron.

Once experiments were complete, rats were euthanized by an overdose of isoflurane *via* inhalation, combined with incising the diaphragm to create a pneumothorax. After perfused with 10% formalin, the dissected brains were fixed with 10% formalin and dehydrated with a 30% sucrose solution, and then sectioned coronally (50 μm) using a cryostat microtome. The brain slices were used to identify electrode recording sites and trajectories that were observed under a microscope. The slices were stained with cresyl violet to verify the detailed structures. The locations of the tips were determined based on the rat brain atlas (Paxinos and Watson, 2013) and were confirmed to be in the mPFC of all rats, including the sub-regions of both prelimbic cortex and infralimbic cortex.

### Experimental Design and Statistical Analyses

Each rat underwent multiple (3–13) test sessions, and during each session, one drug (2MDHX or CY208) at one dose (1, 10, 100, or 10,000 nmol/kg) or vehicle was administered. The vehicle session was always the first, and then our intention was to perform all eight drug conditions in a randomized order. The long-term persistent chronic electrophysiological recording, however, posed a challenge to complete all eight drug conditions, mostly because of the loose microwire electrode array during this long-time process. Eventually one rat completed all drug conditions and even repeated a few, but all others only completed two to five drug conditions (Table 1). To study the neuronal mechanisms, single neurons were recorded from each rat. Number of neurons tested in each drug condition was shown in Table 2. The data were organized based on drug conditions (Vehicle/2MDHX/CY208 @ 1/10/100/10,000 nmol/kg). As detailed below, we used a combination of MATLAB 2017 (MathWorks, Natick, MA, United States), SPSS (IBM, Armonk, NY, United States), and Prism (GraphPad, La Jolla, CA, United States) for data analysis. All data are reported as mean ± SD (*sd* used below for clarity) unless otherwise specified.

**TABLE 1:** Experimental design.

	Number of neurons recorded	2MDHX (nmol/kg)				CY208 (nmol/kg)			
Rat ID		1	10	100	10000	1	10	100	10000
1	45		**x**				**x**		
2	9	**x**		**x**				**x**	
3	41		**x**				**x**		
4	76	**xx**	**xx**	**x**	**x**	**xxx**	**x**	**x**	**x**
5	74		**x**				**x**		
6	69		**x**		**x**		**x**	**x**	**x**

*A total of six rats were used. They were first tested after administration of vehicle (control condition), and then after administration of 2MDHX or CY208 at 1, 10, 100, or 10,000 nmol/kg in a randomized order. Because of the challenge to long-term persistent chronic electrophysiological recording, only one rat (#4) completed all eight drug conditions, and others completed two to five drug conditions. “x” indicates completed drug session. Double or triple “x” indicates repeated sessions after first round of eight drug conditions.*

**TABLE 2:** Data cohort.

Drug condition	Number of neurons (Prospective/Retrospective)	
1 nmol/kg		
2MDHX	35/50	
CY208	30/46	
	*30/46*	*Tested both drugs*
10 nmol/kg		
2MDHX	123/182	
CY208	117/170	
	*117/170*	*Tested both drugs*
100 nmol/kg		
2MDHX	35/50	
CY208	60/94	
	*35/50*	*Tested both drugs*
10,000 nmol/kg		
2MDHX	49/78	
CY208	53/84	
	*49/78*	*Tested both drugs*
	*28/40*	*Tested all eight drug conditions*
	***128/186***	***Total***

*Neuron activity in the PFC first was recorded after administration of vehicle (control condition), and then after administration of 2MDHX or CY208 at 1, 10, 100, or 10,000 nmol/kg. Single neurons recorded during the same drug condition were pooled together for analysis of neuron population dynamics.*

For each neuron, spike counts, i.e., firing rate (FR), were binned around choice (RT, ± 2 s) in each trial, similarly as reported in a previous study (Yang and Mailman, 2018). Each neuron then was classified as either a “prospective-encoding-neuron” or a “retrospective-encoding-neuron” based on the FR before and after the choice behavior during the DAR task, where:


$$N\,e\,u\,r\,o\,n\,T\,y\,p\,e=\left\{ \begin{array}{c} p\,r\,o\,s\,p\,e\,c\,t\,i\,v\,e-e\,n\,c\,o\,d\,i\,n\,g-n\,e\,u\,r\,o\,n,m\,e\,a\,n_{b\,e\,f\,o\,r\,e}>m\,e\,a\,n_{a\,f\,t\,e\,r}\\ r\,e\,t\,r\,o\,s\,p\,e\,c\,t\,i\,v\,e-e\,n\,c\,o\,d\,i\,n\,g-n\,e\,u\,r\,o\,n,m\,e\,a\,n_{b\,e\,f\,o\,r\,e}<m\,e\,a\,n_{a\,f\,t\,e\,r} \end{array}\right.$$

Neuronal sensitivity to correct or error outcome, “neuronal-outcome-sensitivity,” then was scaled by calculating the sensitivity index (*d*′), defined as the ability to distinguish error from correct choice based on the FR, where $d^{{}^{\prime }}=\left(m\,e\,a\,n_{e\,r\,r\,o\,r}-m\,e\,a\,n_{c\,o\,r\,r\,e\,c\,t}\right)/\sqrt{\left(s\,d_{e\,r\,r\,o\,r}^{2}+s\,d_{c\,o\,r\,r\,e\,c\,t}^{2}\right)/2}$.

A positive *d*′ indicates an error outcome sensitivity, whereas a negative *d*′ reflects a correct outcome sensitivity, which classifies neurons into two groups:


$$N\,e\,u\,r\,o\,n\,T\,y\,p\,e=\left\{ \begin{array}{c} c\,o\,r\,r\,e\,c\,t-s\,e\,n\,s\,i\,t\,i\,v\,e-n\,e\,u\,r\,o\,n,d^{{}^{\prime }}<0\\ e\,r\,r\,o\,r-s\,e\,n\,s\,i\,t\,i\,v\,e-n\,e\,u\,r\,o\,n,d^{{}^{\prime }}>0 \end{array}\right.$$

The absolute value of *d*′ reflects the strength of the sensitivity (i.e., higher or lower values suggest greater or less sensitivity, respectively).

For neurons tested both 2MDHX and CY208 at all four doses (1, 10, 100, and 10,000 nmol/kg), the dose for each neuron that led to the largest increase in sensitivity strength was defined as an “optimal” dose for this neuron, whereas the dose that led to the smallest increase or the largest decrease in sensitivity strength was defined as a “detrimental” dose. The median of the optimal and detrimental dose for all these neurons then was calculated and the Wilcoxon matched-pairs signed rank test was used to evaluate if there was a difference between 2MDHX and CY208. The mean sensitivity strength for all neurons also was calculated and repeated ANOVA was used to evaluate whether there was a significant difference among drug conditions (Vehicle/2MDHX/CY208 @ optimal/detrimental dose). The neuron-to-neuron variation, regarding their sensitivity strength, was measured by coefficient of variation (CV) where CV = SD/mean.

Neuronal population dynamics on the dataset that included all recorded neurons then were analyzed. Neurons recorded during the same drug condition (vehicle/2MDHX/CY208 @ 1/10/100/10,000 nmol/kg) were pooled together and three measurements of neuron population dynamics were calculated: percentage, uniformity, and ensemble neuronal sensitivity. *(1)* Percentage. There are two types of neurons based on their sensitivities: “correct-sensitive-neurons” and “error-sensitive-neurons” (see above the definition). The percentage of each group among the whole neuron population was calculated. Fisher’s exact test was used to evaluate whether the percentage differed significantly for drug vs. vehicle. *(2)* Uniformity indicates how homogenous or heterogeneous a group of neurons is regarding their sensitivity. In other words, it measures the diversity of the *d*′ among a group of neurons. To analyze uniformity, a *d*′ proportion distribution of a group of neurons first was calculated using a 0.1-bin and the uniformity then was estimated by the index$H=-\sum_{i=1}^{s}p_{i}\,\ln p_{i}$, where *p*_*i*_ is the proportion in the i*^th^* bin of the *d*′ proportion distribution and s is the number of bins. A low *H* indicates a homogenous population whereas a high *H* indicates a heterogeneous one (Barnes et al., 2005; Thorn et al., 2010; Dorval et al., 2015). ANOVA was used to evaluate whether the uniformity differed significantly after drug administration. *(3)* Ensemble neuronal sensitivity refers to whether a group of neurons integrate their individual neuronal sensitivity to associate with certain (correct/error) information. To analyze the ensemble sensitivity, the median (including its interquartile range) of *d*′ was calculated for correct- and error-sensitive neurons, respectively. Median values farther from zero indicated a higher strength of ensemble sensitivity, whereas those closer to zero indicated a lower strength. The Mann-Whitney test was used to evaluate whether the ensemble sensitivity differed significantly after drug administration.

Finally, we defined a population dynamics index that integrated temporal encoding (prospective and retrospective), outcome sensitivity (correct- and error-sensitive), and three population measurements (percentage, uniformity, and ensemble sensitivity) together. The index was defined as $\sum_{i=1}^{12}F_{i}\,\log\left(V_{i\,\left(d\,r\,u\,g\right)}/V_{i\,\left(v\,e\,h\,i\,c\,l\,e\right)}\right)$, where *V* is the value of a population measurement (percentage, uniformity, or ensemble sensitivity) during the vehicle session or after drug administration, and *F* is the functional index of this population measurement. As shown in Table 3, we defined the functional index of each population measurement as 1 or −1 based on its physiological meaning to best modulate the DAR task performance.

**TABLE 3:** Definition of the integrated population dynamics index $\sum_{i=1}^{12}F_{i}\,\log V_{i\,\left(\mathrm{drug}\right)}/V_{i\,\left(\mathrm{vehicle}\right)}$.

I			F	Functional meaning for modulation of behavioral performance during the DAR task.
T	O	Measurement		
Prospective	Correct	Percentage (%)	**1**	More prospective-correct-neurons to modulate correct outcomes
		Uniformity (H)	**−1**	More homogenous prospective-correct-neurons for correct outcomes
		Ensemble sensitivity (d′)	**1**	Greater correct sensitivity prospectively for correct outcomes
	Error	Percentage (%)	**−1**	Less prospective-error-neurons to limit error outcomes
		Uniformity (H)	**1**	More heterogeneous prospective-error-neurons to limit error outcomes
		Ensemble sensitivity (d′)	**−1**	Less error sensitivity prospectively to limit error outcomes
Retrospective	Correct	Percentage (%)	**1**	More retrospective-correct-neurons for correct outcomes
		Uniformity (H)	**−1**	More homogeneity retrospective-correct-neurons for feedback adjustment
		Ensemble sensitivity (d′)	**1**	Greater correct sensitivity retrospectively for feedback adjustment
	Error	Percentage (%)	**−1**	Less retrospective-error-neurons to limit error outcomes
		Uniformity (H)	**−1**	More homogeneity retrospective-error-neurons for feedback adjustment
		Ensemble sensitivity (d′)	**1**	Greater sensitivity retrospectively for feedback adjustment

*We defined this index to integrate all three measurements of neuron population dynamics (percentage, uniformity, and ensemble sensitivity), and combine them with the consideration of temporal encoding (prospective and retrospective) and event sensitivity (correct- and error-sensitive). V is the value of a population measurement (percentage, uniformity, or ensemble sensitivity) during the vehicle session or after drug administration. F is the functional index of a population measurement, which is either 1 or −1, and indicates an increase (as 1) or a decrease (as −1) of this measurement suggesting a modulation to improve behavioral performance during the DAR. Note the uniformity is calculated by H and the increase of H indicates a decrease of uniformity and vice versa. Therefore, for the uniformity, 1 and −1 indicate an increase (as 1) and a decrease (as −1) of H, which correlates to a decrease (as 1) and an increase (as −1) of uniformity. T, temporal encoding; O, outcome sensitivity.*

## Results

### Dose Response of D_1_ Agonists 2-Methyldihydrexidine and CY208 on Neurons Tested All Four Doses

**FIGURE 2:**
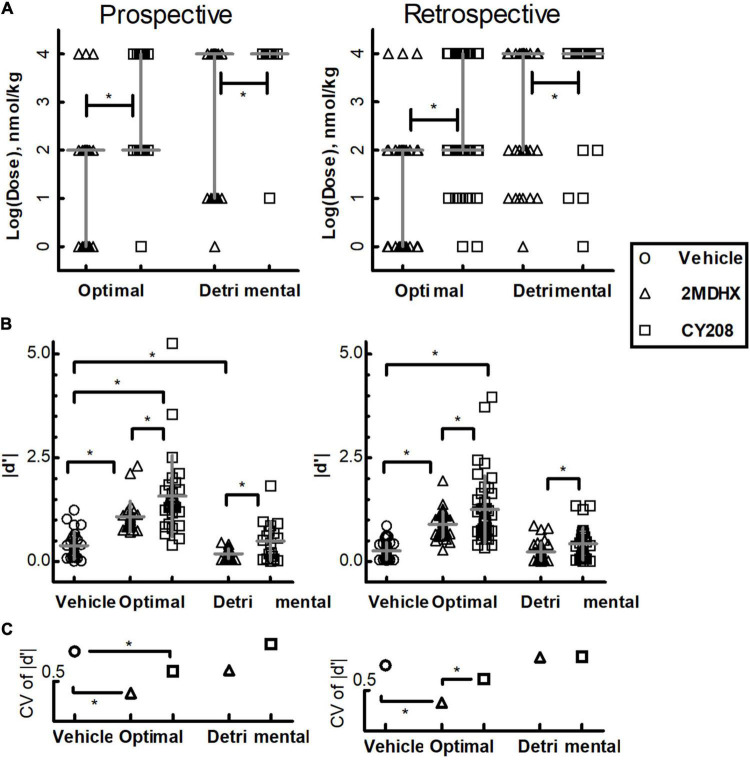
Summary of the effects of D_1_ agonists 2MDHX and CY208 on neurons tested all four doses. **(A)** 2MDHX and CY208 had different optimal and detrimental doses causing maximal increases or decreases in the sensitivity strength of a neuron. See the detailed definitions of optimal and detrimental doses in the Methods. Lines indicate median and interquartile ranges for all prospective- and retrospective-encoding-neurons. Triangles and squares represent each single neuron, and the triangles indicate the 2MDHX and the squares indicate CY208 drug conditions, respectively. Note that both optimal and detrimental doses were lower for 2MDHX compared to CY208 for both prospective- and retrospective-encoding-neurons. *Indicates *P* < 0.05. **(B)** The maximal increase in sensitivity strength at the optimal dose was significantly higher after CY208 administration compared to 2MDHX, and the maximal decrease at the detrimental dose was significantly less by CY208. Lines indicate the average (mean ± SD). Circles indicate the vehicle condition. **(C)** The neuron-to-neuron variation (CV) regarding sensitivity strength was decreased at the optimal dose. Note the decrease in CV was greater by 2MDHX, especially for retrospective-encoding-neurons.

A total of 314 neurons were recorded in the mPFC. An example neuron was showed in Figure 1B, and the summary of all recorded neurons was in Figure 1C and Table 2. Among all recorded neurons, 128 neurons were prospective-encoding-neurons as having higher FR before the choice behavior during the DAR task, whereas 186 neurons were retrospective-encoding-neurons as having higher FR after choice. All neurons were tested during multiple drug conditions, but only 28 prospective-encoding-neurons and 40 retrospective-encoding-neurons were tested for all eight drug conditions (2MDHX/CY208 @ 1/10/100/10,000 nmol/kg). Both 2MDHX and CY208 significantly increased sensitivity strength *|d′|* of these 68 neurons at one of four testing doses, and this “optimal” dose was lower when 2MDHX was administered (prospective-encoding-neurons, *P_2*MDHX,CY*208_* = 0.007; retrospective-encoding-neurons, *P_2*MDHX,CY*208_* < 0.0001; Figure 2A). The increased sensitivity strength was greater for CY208 compared to 2MDHX (Figure 2B and Table 4). Neuron-to-neuron variation, as measured by CV, was decreased by both drugs but the effect was greater for 2MDHX (Figure 2C and Table 4). Both drugs also tended to decrease sensitivity strength at a higher dose, and this “detrimental” dose was lower when 2MDHX was administered (prospective neurons, *P_2*MDHX,CY*208_* = 0.008; retrospective neurons, *P_2*MDHX,CY*208_* = 0.025; Figure 2A). The decrease in sensitivity strength was significant only for prospective-encoding-neurons after 2MDHX administration (Figure 2B and Table 4). There was a trend to increase neuron-to-neuron variation, particularly for CY208 (Figure 2C and Table 4).

**TABLE 4:** Effects of 2MDHX and CY208 on neurons tested all four doses.

D	N		Sensitivity strength |d′|			Variation (CV, %)		
			Vehicle	2MDHX	CY208	Vehicle	2MDHX	CY208
Optimal	Pro		0.38 ± 0.33	1.08 ± 0.38	1.58 ± 0.98	87	35	62
		2MDHX	<0.001*		0.02*	<0.001*		0.097
		CY208	<0.001*	0.02*		0.027	0.097	
	Retro		0.25 ± 0.21	0.89 ± 0.32	1.25 ± 0.81	82	35	65
		2MDHX	<0.001*		0.013*	<0.001*		0.021*
		CY208	<0.001*	0.013*		0.058	0.021*	
Detrimental	Pro		0.38 ± 0.33	0.19 ± 0.12	0.49 ± 0.47	87	64	97
		2MDHX	0.007*		0.016*	0.163		0.19
		CY208	0.391	0.016*		0.802	0.19	
	Retro		0.25 ± 0.21	0.23 ± 0.22	0.42 ± 0.39	82	92	93
		2MDHX	0.665		0.029*	0.925		0.811
		CY208	0.048*	0.029*		0.721	0.811	

Total of 28 prospective-encoding-neurons and 40 retrospective-encoding-neurons were tested for all eight drug conditions (2MDHX/CY208 @ 1/10/100/10,000 nmol/kg). Both 2MDHX and CY208 increased sensitivity strength |d′| and decreased neuron-to-neuron variation (i.e., decreased CV) of these neurons at an “optimal” dose, with CY208 having better effect on |d′| and 2MDHX having better effect on CV. Conversely, at a higher “detrimental” dose, only 2MDHX significantly decreased |d′|. Table shows mean ± SD on the first row of each section and p-values of repeated ANOVA on the second and third rows. D, dose (nmol/kg); N, neuron type (prospective-neuron, retrospective-neuron); Pro: prospective; Retro: retrospective. *Indicates significance.

### Dose Response of 2-Methyldihydrexidine on Pooled Neuron Population

**FIGURE 3:**
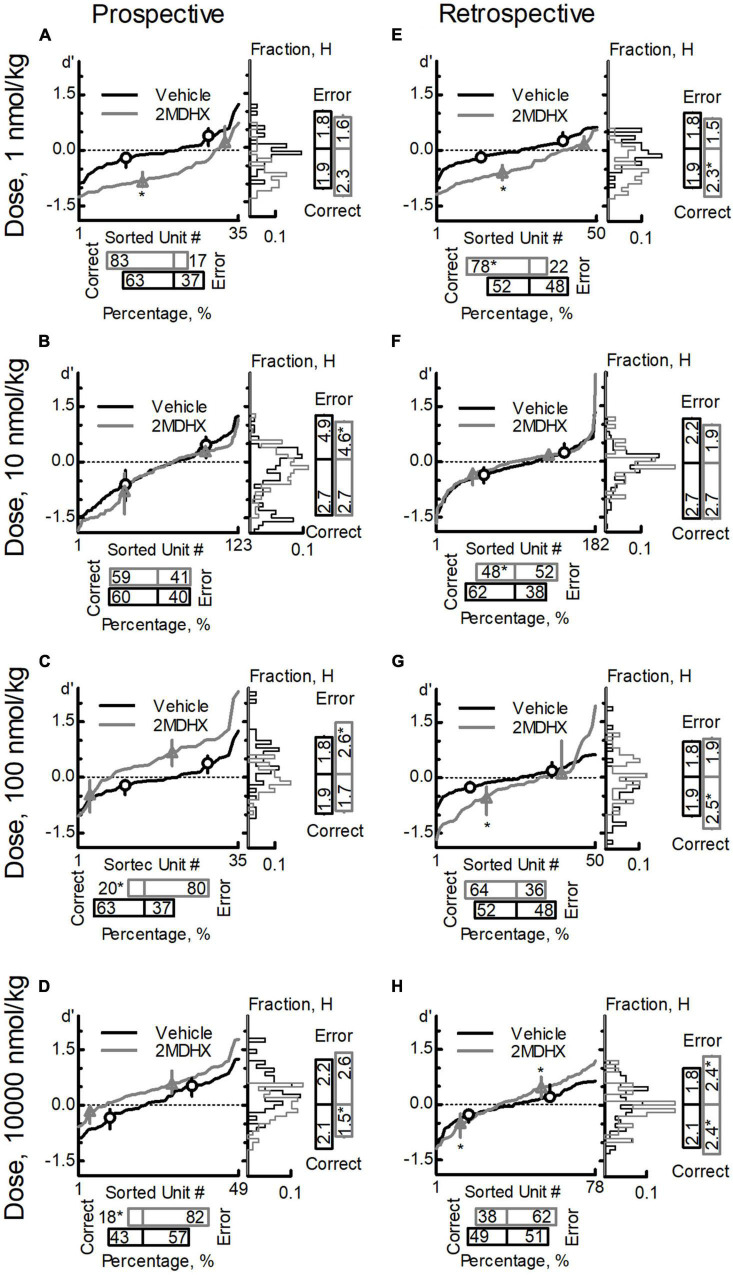
Effects of 2MDHX on neuron population dynamics engaged in the sWM-related DAR task. Three measurements of population dynamics (ensemble sensitivity, uniformity, and percentage) were evaluated for the effects of 2MDHX at four doses: 1 nmol/kg **(A,E)**, 10 nmol/kg **(B,F)**, 100 nmol/kg **(C,G)**, and 10,000 nmol/kg **(D,H)**. All calculations are based on the neuronal-outcome-sensitivity (*d*′) of each neuron in the group (refer to the Methods for detailed definitions of each measurement). The lines in each panel show the value of *d*′ for each neuron. The circle (vehicle) and up-pointed-triangle (2MDHX) on the lines indicate the median and interquartile range of the correct- or error-sensitive-neurons, respectively, which is the indicator of ensemble sensitivity for the population. A histogram of the *d*′ distribution is shown on the right of each panel. The vertical bars on the right indicate the uniformity level of a population (H), and the horizontal bars at the bottom indicate the percentage of correct- or error-sensitive-neurons. *Indicates *P* < 0.05 for the comparison between vehicle (black, circle) and 2MDHX (gray, up-pointed-triangle) conditions. **(A–D)** show the results for prospective-encoding-neurons and **(E,F)** show the results for retrospective-encoding-neurons. Note the variable effects of different doses of 2MDHX on the three parameters.

We pooled neurons tested in the same drug condition together and then analyzed drug effects on the neuron population dynamics comparing to vehicle condition. For prospective-encoding-neurons, 2MDHX increased the percentage of correct-sensitive-neurons at 1 nmol/kg, but it was not statistically significant (Figure 3A and Table 5). At higher doses, 2MDHX significantly decreased the percentage of correct-sensitive-neurons (10–10,000 nmol/kg; Figures 3B–D and Table 5). Uniformity of the correct-sensitive-neurons, as measured by H, did not change at lower doses (1–100 nmol/kg; Figures 3A–C and Table 5), but became slightly more homogeneous (i.e., decreased H) at 10,000 nmol/kg (Figure 3D and Table 5). The ensemble sensitivity of the correct-neurons, as measured by median of *d*′, was increased at 1 nmol/kg (Figure 3A and Table 5), but at higher doses the increase became not statistically significant (10–10,000 nmol/kg; Figures 3B–D and Table 5). For error-sensitive-neurons, their uniformity (H) was not changed at 1 nmol/kg (Figure 3A and Table 5) but became more homogeneous (i.e., decreased H) at 10 nmol/kg (Figure 3B and Table 5). At higher doses, however, the uniformity became more heterogeneous (i.e., increased H; 100–10,000 nmol/kg; Figures 3C,D and Table 5). The ensemble sensitivity (*d*′) of the error-neurons tended to decrease at lower doses (1–10 nmol/kg; Figures 3A,B and Table 5), but increased slightly at higher doses (100–10,000 nmol/kg; Figures 3C,D and Table 5).

**TABLE 5:** Effects of 2MDHX on neuron population dynamics.

Neuron Type		Dose	Percentage (%)			Uniformity (H)			Ensemble sensitivity (d′)		
			Vehicle	2MDHX	*p*	Vehicle	2MDHX	*p*	Vehicle	2MDHX	*p*
Prospective	Correct	1	63	83	0.1055	1.90 ± 0.03	2.27 ± 0.01	0.0686	−0.21 (−0.47, −0.09)	−0.84 (−0.99, −0.60)	<0.0001*
		10	60	59	1	2.69 ± 0.003	2.70 ± 0.01	0.9891	−0.60 (−1.0, −0.22)	−0.77 (−1.4, −0.30)	0.0938
		100	63	20	0.0006*	1.90 ± 0.03	1.75 ± 0.09	0.6644	−0.21 (−0.47, −0.09)	−0.47 (−0.93, −0.08)	0.2732
		10,000	43	18	0.0152*	2.11 ± 0.02	1.52 ± 0.05	0.0463*	−0.34 (−0.63, −0.11)	−0.15 (−0.49, −0.04)	0.2393
	Error	1	37	17	0.1055	1.84 ± 0.04	1.56 ± 0.10	0.46	0.38 (0.12, 0.58)	0.22 (0.05, 0.63)	0.5107
		10	40	41	1	2.20 ± 0.01	1.89 ± 0.01	0.0425*	0.47 (0.12, 0.68)	0.30 (0.17, 0.39)	0.0642
		100	37	80	0.0006*	1.84 ± 0.04	2.55 ± 0.02	0.0071*	0.38 (0.12, 0.58)	0.68 (0.31, 1.0)	0.0586
		10,000	57	82	0.0152*	2.21 ± 0.02	2.56 ± 0.01	0.0503	0.53 (0.25, 0.73)	0.58 (0.32, 0.93)	0.2811
Retrospective	Correct	1	52	78	0.0113*	1.86 ± 0.02	2.30 ± 0.01	0.0112*	−0.25 (−0.42, −0.09)	−0.63 (−0.84, −0.44)	<0.0001*
		10	62	48	0.0114*	2.39 ± 0.01	2.32 ± 0.01	0.5123	−0.34 (−0.56, −0.15)	−0.31 (−0.61, −0.20)	0.8069
		100	52	64	0.3111	1.86 ± 0.02	2.49 ± 0.02	0.0017*	−0.22 (−0.36, −0.08)	−0.50 (−0.96, −0.21)	0.0012*
		10,000	49	38	0.1055	2.06 ± 0.01	2.40 ± 0.01	0.0445*	−0.25 (−0.46, −0.12)	−0.47 (−0.86, −0.23)	0.0142*
	Error	1	48	22	0.0113*	1.77 ± 0.02	1.47 ± 0.05	0.2563	0.22 (0.11, 0.44)	0.14 (0.05, 0.33)	0.2202
		10	38	52	0.0114*	1.99 ± 0.01	1.89 ± 0.01	0.4015	0.26 (0.12, 0.50)	0.20 (0.10, 0.32)	0.0663
		100	48	36	0.3111	1.77 ± 0.02	1.91 ± 0.05	0.5954	0.22 (0.11, 0.44)	0.15 (0.10, 1.03)	0.8688
		10,000	51	62	0.1055	1.79 ± 0.01	2.38 ± 0.01	<0.0001*	0.23 (0.12, 0.55)	0.49 (0.20, 0.76)	0.0072*

Neurons tested in the same dose were pooled together for analyzing 2MDHX’s effects on the population dynamics comparing to vehicle. Percentage of correct- or error-sensitive-neuron, uniformity (H) and ensemble (d′) of the population, regarding its neuronal-outcome-sensitivity were examined (see Methods for details). The p-values were from Fisher’s exact test (Percentage), ANOVA (Uniformity), and Mann-Whitney test (Ensemble sensitivity). Doses are in nmol/kg. *Indicates significance.

For retrospective-encoding-neurons, 2MDHX increased the percentage of correct-sensitive-neurons at 1 nmol/kg (Figure 3E and Table 5), decreased it at 10 nmol/kg (Figure 3F and Table 5), and had no significant effects at higher doses (100–10,000 nmol/kg; Figures 3G,H and Table 5). Uniformity (H) of the correct-sensitive-neurons became more heterogeneous (i.e., increased H) at most tested doses (1, 100, and 10,000 nmol/kg; Figures 3E,G,H and Table 5), except at 10 nmol/kg where the effect was not significant (Figure 3F and Table 5). The ensemble sensitivity (*d*′) of the correct-neurons increased at most tested doses (1, 100, and 10,000 nmol/kg; Figures 3E,G,H and Table 5), except at 10 nmol/kg where the effect was not significant (Figure 3F and Table 5). For error-sensitive-neurons, their uniformity (H) tended to be more homogeneous (i.e., decreased H) at lower doses (1–10 nmol/kg; Figures 3E,F and Table 5), but became more heterogeneous (i.e., increased H) at higher doses (100–10,000 nmol/kg; Figures 3G,H and Table 5). The ensemble sensitivity (*d*′) of the error-neurons did not change significantly at lower doses (1–100 nmol/kg; Figures 3E–G and Table 5), but was increased at 10,000 nmol/kg (Figure 3H and Table 5).

### Dose Response of CY208 on Pooled Neuron Population

**FIGURE 4:**
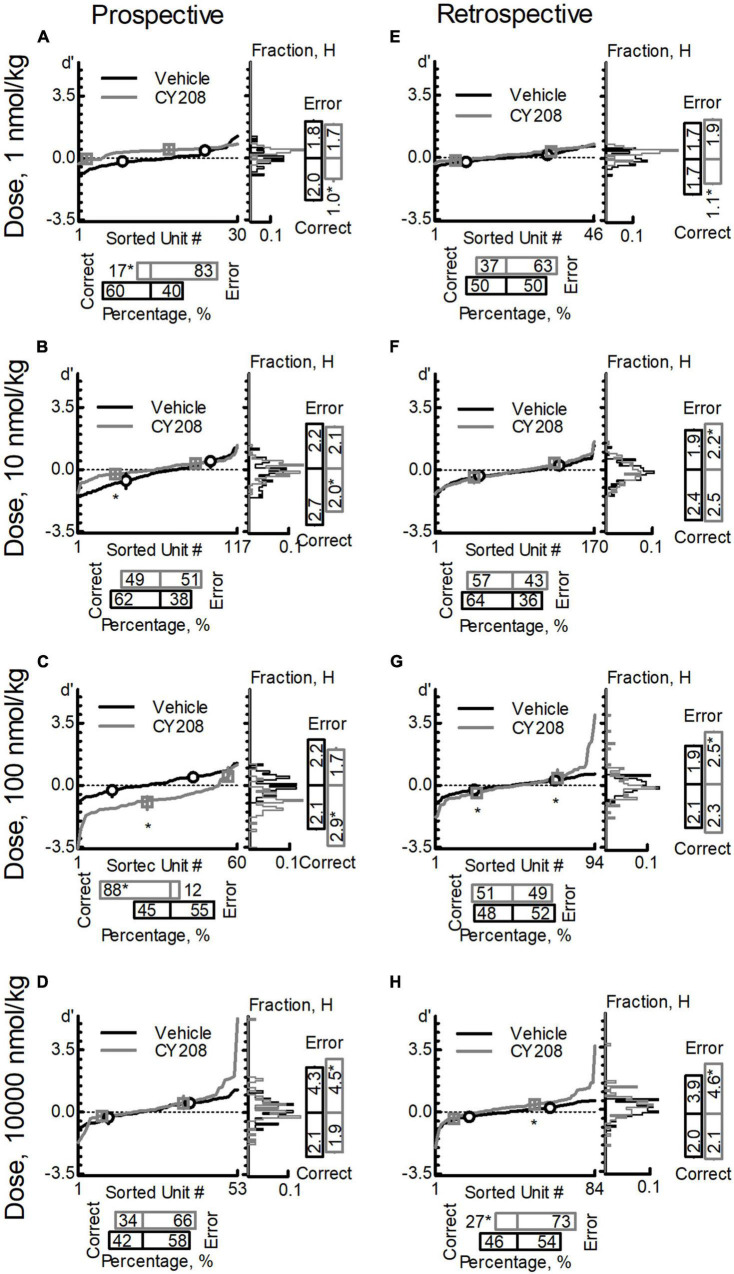
Effects of CY208 on neuron population dynamics engaged in the sWM-related DAR task. Similar to Figure 3, three measurements of population dynamics (ensemble sensitivity, uniformity, and percentage) were evaluated for the effects of CY208 at four doses: 1 nmol/kg **(A,E)**, 10 nmol/kg **(B,F)**, 100 nmol/kg **(C,G)**, and 10,000 nmol/kg **(D,H)**. All calculations are based on the neuronal-outcome-sensitivity (*d*′) of each neuron in the group (refer to the Methods for the detailed definition of each measurement). The lines in each panel show the value of *d*′ for each neuron. The circle (vehicle) and square (CY208) on the lines indicate the median and interquartile range of the correct- or error-sensitive-neurons, respectively, an indicator of the ensemble sensitivity for the population. The histogram of *d*′ distribution is shown on the right of each panel. The vertical bars on the right indicate the uniformity level of a population (H), and the horizontal bars at the bottom indicate the percentage of correct- or error-sensitive-neurons. *Indicates *P* < 0.05 for the comparison between vehicle (black, circle) and CY208 (gray, square) conditions. **(A–D)** show the results for prospective-encoding-neurons and **(E,F)** show the results for retrospective-encoding-neurons. Note the variable effects of CY208 at different dose on three measurements.

For prospective-encoding-neurons, CY208 decreased the percentage of correct-sensitive-neurons at most tested doses (1, 10, and 10,000 nmol/kg; Figures 4A,B,D and Table 6), and only increased it at 100 nmol/kg (Figure 4C and Table 6). Uniformity (H) of the correct-sensitive-neurons became more homogenous (i.e., decreased H) at most tested doses (1, 10, and 10,000 nmol/kg; Figures 4A,B,D and Table 6), excepted at 100 nmol/kg where it became more heterogeneous (i.e., increased H; Figure 4C and Table 6). The ensemble sensitivity (*d*′) of correct-neurons was decreased at most tested doses, especially 10 nmol/kg (1, 10, and 10,000 nmol/kg; Figures 4A,B,D and Table 6), except at 100 nmol/kg where it was increased (Figure 4C and Table 6). For error-sensitive-neurons, their uniformity (H) was not changed at lower doses (1–100 nmol/kg; Figures 4A–C and Table 6) but became more heterogeneous (i.e., increased H) at 10,000 nmol/kg (Figure 4D and Table 6). The ensemble sensitivity (*d*′) of error-neurons did not change at any tested dose (Figures 4A–D and Table 6).

**TABLE 6:** Effects of CY208 on neuron population dynamics.

Neuron Type		Dose	Percentage (%)			Uniformity (H)			Ensemble sensitivity (d′)		
			Vehicle	CY208	*p*	Vehicle	CY208	*p*	Vehicle	CY208	*p*
Prospective	Correct	1	60	17	0.0012*	1.99 ± 0.02	0.95 ± 0.14	0.0419*	−0.22 (−0.50, −0.11)	−0.09 (−0.27, −0.09)	0.1679
		10	62	49	0.0655	2.69 ± 0.004	2.04 ± 0.01	<0.0001*	−0.62 (−1.1, −0.23)	−0.25 (−0.44, −0.13)	<0.0001*
		100	45	88	<0.0001*	2.07 ± 0.02	2.87 ± 0.01	<0.0001*	−0.22 (−0.59, −0.09)	−0.88 (−1.3, −0.58)	<0.0001*
		10,000	42	34	0.548	2.10 ± 0.02	1.88 ± 0.03	0.3378	−0.28 (−0.63, −0.12)	−0.21 (−0.50, −0.15)	0.9242
	Error	1	40	83	0.0012*	1.82 ± 0.05	1.67 ± 0.02	0.58	0.39 (0.12, 0.59)	0.48 (0.38, 0.56)	0.3899
		10	38	51	0.0655	2.20 ± 0.01	2.06 ± 0.01	0.3513	0.47 (0.11, 0.68)	0.34 (0.23, 0.48)	0.2478
		100	55	12	<0.0001*	2.23 ± 0.02	1.75 ± 0.09	0.1628	0.52 (0.25, 0.72)	0.55 (0.42, 1.1)	0.2262
		10,000	58	66	0.548	2.21 ± 0.02	2.61 ± 0.01	0.0268*	0.53 (0.25, 0.73)	0.52 (0.21, 0.92)	0.7383
Retrospective	Correct	1	50	37	0.293	1.67 ± 0.02	1.15 ± 0.03	0.0193*	−0.20 (−0.34, −0.08)	−0.12 (−0.20, −0.07)	0.0848
		10	4	57	0.2677	2.40 ± 0.01	2.46 ± 0.004	0.5873	−0.35 (−0.60, −0.17)	−0.43 (−0.76, −0.20)	0.1396
		100	48	51	0.7706	2.07 ± 0.01	2.30 ± 0.0	0.1115	−0.25 (−0.49, −0.11)	−0.45 (−0.72, −0.15)	0.0181*
		10,000	46	27	0.0161*	2.05 ± 0.01	2.13 ± 0.03	0.6988	−0.25 (−0.45, −0.11)	−0.35 (−0.54, −0.14)	0.4145
	Error	1	50	63	0.293	1.75 ± 0.02	1.93 ± 0.01	0.355	0.19 (0.11, 0.45)	0.38 (0.16, 0.51)	0.2036
		10	96	43	0.2677	1.94 ± 0.01	2.23 ± 0.01	0.0209*	0.23 (0.12, 0.52)	0.36 (0.17, 0.61)	0.0815
		100	52	49	0.7706	1.89 ± 0.004	2.54 ± 0.01	<0.0001*	0.27 (0.13, 0.47)	0.41 (0.13, 0.87)	0.0464*
		10,000	54	73	0.0161*	1.87 ± 0.01	2.45 ± 0.01	<0.0001*	0.27 (0.12, 0.51)	0.44 (0.25, 0.71)	0.0058*

Similar to Table 5, neurons tested in the same dose were pooled together for analyzing CY208’s effects on the population dynamics comparing to vehicle. Percentage of correct- or error-sensitive-neuron, uniformity (H) and ensemble (d′) of the population were examined (see Methods for details). The p-values were from Fisher’s exact test (Percentage), ANOVA (Uniformity), and Mann-Whitney test (Ensemble sensitivity). Dose are expressed in nmol/kg. *Indicates significance.

For retrospective-encoding-neurons, CY208 at lower doses had no significant effect on the percentage of correct-sensitive-neurons (1–100 nmol/kg; Figures 4E–G and Table 6), but decreased it at 10,000 nmol/kg (Figure 4H and Table 6). Uniformity (H) of the correct-sensitive-neurons became more homogeneous (i.e., decreased H) at 1 nmol/kg (Figure 4E and Table 6), but at higher doses it became heterogeneous (i.e., increased H) though not significantly (10–10,000 nmol/kg; Figures 4F–H and Table 6). The ensemble sensitivity (*d*′) of correct-neurons tended to decrease at 1 nmol/kg (Figure 4E and Table 6), but was increased at higher doses, especially 100 nmol/kg (10–10,000 nmol/kg; Figures 4F–H and Table 6). For error-sensitive-neurons, their uniformity (H) became more homogeneous (i.e., decreased H) at 1 nmol/kg though not significantly (Figure 4E and Table 6), but at higher doses it became more heterogeneous (i.e., increased H; 10–10,000 nmol/kg; Figures 4F–H and Table 6). The ensemble sensitivity (*d*′) for error-neurons was increased, but only achieved significance at higher doses (Figures 4E–H and Table 6).

### Comparison Between 2-Methyldihydrexidine and CY208

To investigate whether there was a difference between 2MDHX and CY208 affecting neuron population dynamics, we analyzed the neurons that were tested for both drugs at a dose. For prospective-encoding-neurons, there was more correct-sensitive-neurons after administration of 2MDHX at lower doses compared to CY208 (1–10 nmol/kg, Table 7), whereas at higher doses, it was after administration of CY208 that there was more correct-sensitive-neurons (100–10,000 nmol/kg, Table 7). Uniformity (H) of the correct-sensitive neurons was more homogenous (i.e., decreased H) after administration of CY208 at lower doses compared to 2MDHX (1–10 nmol/kg, Table 7), whereas at higher doses, it was after administration of 2MDHX that uniformity of the correct-sensitive neurons was more homogenous (100–10,000 nmol/kg, Table 7). The ensemble sensitivity (*d*′) of correct-neurons was higher after administration of 2MDHX at lower doses compared to CY208 (1–10 nmol/kg, Table 7), whereas at higher doses, it was after CY208 administration that the ensemble sensitivity of correct-neurons trended higher (100–10,000 nmol/kg, Table 7). For error-sensitive neurons, uniformity (H) was more heterogeneous (i.e., increased H) after administration of CY208 at lower doses compared to 2MDHX (1–10 nmol/kg, Table 7), whereas at higher doses, it was after 2MDHX administration that uniformity of error-sensitive neurons was more heterogeneous (100–10,000 nmol/kg, Table 7). Regarding the ensemble sensitivity (*d*′) of the error-neurons, there was no significant difference between 2MDHX and CY208 at all tested doses (Table 7).

**TABLE 7:** Compare effects of 2MDHX with CY208 on neuron population dynamics.

Neuron Type		Dose	Percentage (%)			Uniformity (H)			Ensemble sensitivity (d′)		
			2MDHX	CY208	*p*	2MDHX	CY208	*p*	2MDHX	CY208	*p*
Prospective	Correct	1	90	17	<0.0001*	2.24 ± 0.02	0.95 ± 0.10	0.0089*	−0.85 (−0.99, −0.65)	−0.09 (−0.27, −0.09)	0.0009*
		10	62	49	0.0482*	2.70 ± 0.0	2.04 ± 0.01	<0.0001*	−0.77 (−1.4, −0.30)	−0.25 (−0.44, −0.13)	<0.0001*
		100	20	89	<0.0001*	1.75 ± 0.09	2.91 ± 0.02	0.0055*	−0.46 (−0.93, −0.08)	−0.93 (−1.33, −0.52)	0.0707
		10,000	18	31	0.2399	1.52 ± 0.05	1.96 ± 0.03	0.1644	−0.15 (−0.49, −0.04)	−0.22 (−0.86, −0.18)	0.2105
	Error	1	10	83	<0.0001*	1.10 ± 0.11	1.67 ± 0.02	0.1919	0.60 (0.06, 0.73)	0.48 (0.38, 0.56)	0.6031
		10	38	51	0.0482*	1.74 ± 0.01	2.06 ± 0.01	0.0355*	0.29 (0.17, 0.37)	0.34 (0.23, 0.48)	0.0631
		100	80	11	<0.0001*	2.55 ± 0.02	1.39 ± 0.16	0.0394*	0.68 (0.31, 1.01)	0.48 (0.36, 0.94)	0.9319
		10,000	82	69	0.2399	2.56 ± 0.01	2.61 ± 0.01	0.7279	0.58 (0.32, 0.93)	0.54 (0.18, 0.93)	0.6371
Retrospective	Correct	1	76	37	0.0003*	2.26 ± 0.01	1.15 ± 0.03	<0.0001*	−0.66 (−0.85, −0.49)	−0.12 (−0.20, −0.07)	<0.0001*
		10	46	57	0.0506	2.32 ± 0.01	2.46 ± 0.004	0.2125	−0.31 (−0.62, −0.18)	−0.43 (−0.76, −0.20)	0.1753
		100	64	52	0.3111	2.49 ± 0.02	2.36 ± 0.02	0.5021	−0.50 (−0.96, −0.21)	−0.55 (−0.82, −0.29)	0.9191
		10,000	38	28	0.2343	2.40 ± 0.01	2.16 ± 0.03	0.2742	−0.47 (−0.86, −0.23)	−0.36 (−0.56, −0.14)	0.162
	Error	1	24	63	0.0003*	1.47 ± 0.05	1.93 ± 0.01	0.0824	0.14 (0.05, 0.33)	0.38 (0.16, 0.51)	0.0366*
		10	54	43	0.0506	1.79 ± 0.01	2.23 ± 0.01	0.0004*	0.19 (0.10, 0.29)	0.36 (0.17, 0.61)	<0.0001*
		100	36	48	0.3111	1.91 ± 0.05	2.67 ± 0.02	0.0073*	0.15 (0.10, 1.03)	0.67 (0.28, 1.15)	0.0732
		10,000	62	72	0.2343	2.38 ± 0.01	2.46 ± 0.01	0.5311	0.49 (0.20, 0.76)	0.46 (0.25, 0.74)	0.8679

To investigate whether 2MDHX and CY208 affected neuron population dynamics differently, we analyzed the neurons that were tested for both drugs at a same dose. Similar to Tables 5, 6, percentage of correct- or error-sensitive-neuron, uniformity (H) and ensemble (d′) of the population were examined (see Methods for details). The p-values were from Fisher’s exact test (Percentage), ANOVA (Uniformity), and Mann-Whitney test (Ensemble sensitivity). Doses are in nmol/kg. *Indicates significance.

For retrospective-encoding-neurons, there were more correct-sensitive-neurons after administration of 2MDHX at 1 nmol/kg compared to CY208 (Table 7), but not at any other tested dose (10–10,000 nmol/kg, Table 7). Uniformity (H) of the correct-sensitive neurons was more homogenous (i.e., decreased H) after administration of CY208 at 1 nmol/kg compared to 2MDHX (Table 7), but not at any other tested dose (10–10,000 nmol/kg, Table 7). The ensemble sensitivity (*d*′) of the correct-neurons was higher after administration of 2MDHX at 1 nmol/kg compared to CY208 (Table 7), but not at any other tested dose (10–10,000 nmol/kg, Table 7). For error-sensitive-neurons, their uniformity (H) was more homogenous (i.e., decreased H) after administration of 2MDHX compared to CY208, especially at 10 and 100 nmol/kg (Table 7). The ensemble sensitivity (*d*′) for error-neurons was higher after CY208 administration compared to 2MDHX, especially at lower doses (Table 7).

**FIGURE 5:**
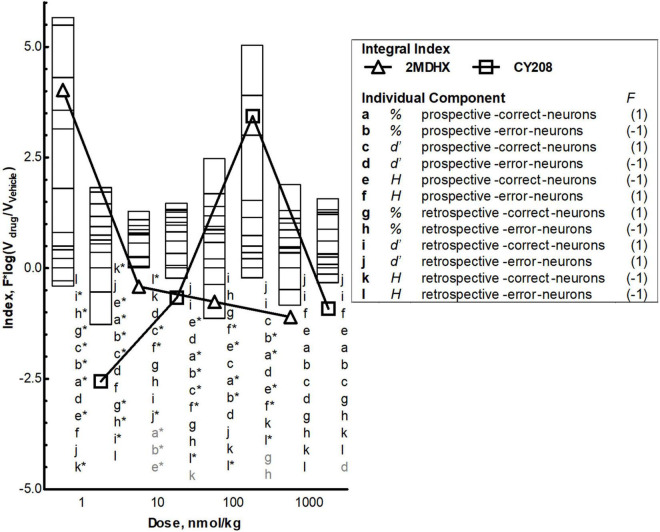
Integrated effects and comparison between 2MDHX and CY208. An integrated population dynamics index, $\sum_{i=1}^{12}F_{i}\,\log\left(V_{i\,\,\left(d\,r\,u\,g\right)}/V_{i\,\,\left(v\,e\,h\,i\,c\,l\,e\right)}\right)$, was defined to combine all three measurements [percentage (abgh), uniformity (efkl), and ensemble sensitivity (cdij)] with the temporal encoding [prospective (a–f) and retrospective (g–l)] and event sensitivity [correct (acegik) and error (bdfhjl)]. These 12 letters (a–l) represent each component of the integrated index, and the number 1/–1 next to the letter indicates the value of functional index (F) for this component (refer to the Methods for details). Stacked bars show the value of each component at four doses (1, 10, 100, and 10,000 nmol/kg), and triangles and squares show the value of integral index, with triangles indicate the 2MDHX and squares indicate CY208, respectively. The letters next to the bars indicate the order of the stacked bars, and the gray colored letters indicate that the value of these components was too small to be illustrated by the stacked bar. The * next to the letter indicates *P* < 0.05 for the comparison between 2MDHX and CY208 for this component. Note the inverted-*U* curve of the integral index for CY208, the higher integral index values for 2MDHX compared to CY208, and the shift to lower doses of the integral index curve for 2MDHX compared to CY208.

Finally, we calculated a population dynamics index that integrates three measurements (percentage, uniformity, and ensemble sensitivity) together and combines outcome sensitivity and temporal encoding. Both 2MDHX and CY208 had dose-dependent effects on population index, with lower doses (2MDHX, 1 nmol/kg; CY208, 100 nmol/kg; Figure 5) having a positive impact (i.e., higher value of the index) that diminished at higher doses (2MDHX, 10, 100, and 10000 nmol/kg; CY208, 10000 nmol/kg). The results also suggested that 2MDHX had a larger impact compared to CY208 (2MDHX vs. CY208 = 4.0 vs. 3.4), implying 2MDHX had a higher efficiency. In addition, the index dose response curve of 2MDHX was shifted to lower doses compared to CY208, suggesting a higher potency for 2MDHX.

## Discussion

### D_1_ Dose-Dependency of Prefrontal Cortex Neuronal Activities

This study tested D_1_ agonists in a sWM-related T-maze task. By using a wide range of log-spaced doses, we assessed the dose-dependency of PFC neuronal population activities related to sWM. There were complicated dose-response effects at the neuron population level, but overall it followed an inverted-U curve, consistent with the dose response at the single neuron level reported previously using other D_1_ agents and cognitive-related tasks (Vijayraghavan et al., 2007; Wang et al., 2019). Our data support the hypothesis that D_1_ dose-dependent effects on cognition were represented not only by single neuron activities, but also by neuron population dynamics in the PFC that eventually propagates to the behavioral level (Yang et al., 2021).

Two prior studies examined the D_1_ dose-dependency at the single neuron level in the non-human primate PFC that maintain a persistent firing to represent active maintenance of the sWM. Their overall conclusion was that D_1_ agonists improve single neuron activities through a “sculpting action” (Vijayraghavan et al., 2007; Wang et al., 2019). In our experimental paradigm, the neuronal population activities represent strategic encoding of the choice behavior in the T-maze task that reflects flexible updating of the sWM (Yang and Mailman, 2018). Our data showed that the drug effects on outcome-sensitivity of the group of neurons tested all four doses, although was from only one animal, paralleled this “sculpting action,” such that increased sensitivity at an “optimal” dose was decreased at higher, “detrimental,” doses. The inconsistencies between the two compounds, however, are intriguing. Specifically, CY208 was superior at improving sensitivity strength at an optimal dose and maintaining it at higher doses, but 2MDHX deceased neuron-to-neuron variation more. These results highlight the importance for examining dose-dependency not only at the single neuron, but also neuron population level, as neuron-to-neuron variation reflects neuron population dynamics. Moreover, the fact that the PFC contains dynamic neural activities (Yang and Mailman, 2018; De Falco et al., 2019; Kaminski and Rutishauser, 2019) suggests that the pattern of dose-dependency at the single neuron level is dissimilar from that at the neuron population level. Indeed, compared to single neuron activities reported previously (Vijayraghavan et al., 2007; Wang et al., 2019), neuron population dynamics reported in current study are dose-dependent, but their pattern is relatively irregular and does not match the typical inverted-U curves.

In the current study, we focused on three aspects of neuron population dynamics: percentage; uniformity; and ensemble sensitivity. We pooled all recorded neurons together and analyzed. Our hypothesis was that optimal doses of D_1_ agonists will increase the population of prospective-encoding-correct-sensitive-neurons with strengthened uniformity and ensemble sensitivity and decrease the population of prospective-encoding-error-sensitive-neurons with decreased uniformity and ensemble sensitivity, leading to a greater probability for a correct outcome. Concomitantly, D_1_ agonists also should increase the uniformity and ensemble sensitivity of retrospective-encoding-neurons, with either correct or error sensitivity, leading to better feedback adjustment. Our results were partially consistent, albeit the dose response curves are more complex. It is puzzling that none of the measurements at the neuron population level showed a clear inverted-U/biphasic dose response curve as occurred at the single neuron level (Vijayraghavan et al., 2007; Wang et al., 2019). It is possible that the modest data sample (minimum of n = 30 neurons) was insufficient to detect this. Another possibility is that the high plasticity in the PFC (De Falco et al., 2019; Singh et al., 2019) results in highly variable dose-response curves, although this seems unlikely because all drug tests were performed after rats were well trained for the task. The third explanation is that diverse single neuron activities in the PFC (Yang and Mailman, 2018; De Falco et al., 2019; Kaminski and Rutishauser, 2019) could contribute to this complicated neural modulation at the population level. Our data indeed showed different neuron population among individual rats. This difference potentially may represent different cognitive ability of each individual rats for performing DAR task. It is noteworthy that further analysis showed that although there were irregular patterns over the dose range for each individual measurement, integrating all three measures for every neuron population revealed a relatively clear dose-dependency, that is, the integrated population index was higher at an “optimal” dose and became lower at a higher “detrimental” dose. Although the findings of the current study are complicated, they are the first step in creating a useful model for D_1_-related dose-dependent regulation of cognition. Future studies should focus on additional aspects of neuron population dynamics and other behavioral tasks evaluating different domains of WM.

### Differences Between 2-Methyldihydrexidine and CY203

There were some striking differences between the effects of 2MDHX and CY208 on PFC neuronal population activities during the sWM task. Overall, 2MDHX had greater effects on integrated neuron population dynamics since its maximum index was higher (implying better efficiency), and the dose response curve of the index was shifted to lower doses (suggesting higher potency). The higher potency of 2MDHX was consistent for the group of neurons tested all four doses, such that the optimal dose for improving neuronal sensitivity was lower after 2MDHX administration compared to CY208. The efficiency of 2MDHX, however, was not greater for this group of neurons. The sensitivity strength was improved less by 2MDHX compared to CY208, although 2MDHX reduced the neuron-to-neuron variation more. Both 2MDHX and CY208 had variable effects on neuron population dynamics and neither showed consistent improvement on the three measurements (percentage; uniformity; and ensemble strength of sensitivity). The overall impression was that 2MDHX was better at increasing the percentage of correct-sensitive-neurons and ensemble sensitivity, whereas CY208 was better at modulating uniformity. For retrospective encoding that may represent a perdurance of activity related to previous choice and could be potentially important for neural feedback adjustment, 2MDHX was better at modulating uniformity, and CY208 was better at increasing the ensemble sensitivity. How these differences at the neuronal population level propagate to behavior is an unsolved question, but it could be proposed that the difference between 2MDHX and CY208, regarding their effects on the time to make the choice in the DAR task as reported in our previous publication (Yang et al., 2021), is one of the behaviors manifested from these neuronal population differences.

It is unclear whether these dissimilarities were due to ligand differences in D_1_R signaling bias or some other mechanism. In many pharmacological studies, the drug doses used are likely to engage secondary targets (Lee et al., 2014). We believe off-target effects in the current study can be ruled out by the sensitivity of PFC D_1_Rs that allowed the use of very low drug doses. Fractional receptor occupancy would be very low based on the apparent affinities of 2MDHX and CY208 for the D_1_R (Markstein et al., 1992; Knoerzer et al., 1995). Indeed, the doses used in the current study were far lower than those from an earlier report that concluded such effects occurred *via* the D_1_R (Isacson et al., 2004).

Our working hypothesis is that D_1_R functional selectivity is the major mechanism underlying these dissimilarities between 2MDHX and CY208, i.e., 2MDHX has full intrinsic activity at cAMP and > > 100% at β-arrestin signaling, whereas CY208 has high intrinsic activity at cAMP signaling, but partial agonist activity at β-arrestin signaling (Yang et al., 2021). The difference between 2MDHX and CY208 could be interpreted in several ways: (1) D_1_-mediated β-arrestin signaling has a major influence on the potency of the dose-response since higher activity (by 2MDHX) leads to higher potency (i.e., the optimal dose for improving neuronal-sensitivity was lower); (2) cAMP signaling may have more influence on the efficiency of the dose-response since higher activity (by CY208) leads to higher efficiency (i.e., more improvement on neuronal-sensitivity); (3) dose-dependency at the neuron population level in the PFC is a result of balancing cAMP and β-arrestin signaling; and/or (4) differential bias at another signaling pathway is involved.

Our data suggest but does not provide direct evidence that D_1_-mediated cAMP and/or β-arrestin signaling cooperate to regulate the dose-dependency of sWM-related neural activities in the PFC. Although both pathways are important modulators of dopamine function (Vijayraghavan et al., 2007; Wang et al., 2019; Yang et al., 2021), other signaling pathways modulated by the D_1_Rs also may contribute. In addition, factors other than signaling bias (e.g., pharmacokinetics or metabolite formation) can complicate the results and account for the dose-dependent response of PFC neural activities. Moreover, there are no known ligands that have marked selectivity for the D_1_ vs. the highly homologous D_5_ dopamine receptor. We have used the term “D_1_,” but are keenly aware that D_5_ mechanisms may contribute. Future studies should consider alternate models, other aspects of neural activity, and advanced techniques to gain improved insight into how functional selectivity may modify D_1_-related dose-dependency and relate to enhanced therapeutics.

### Conclusion: Clinical Implications

Marked cognitive improvement by D_1_ agonists has been a consistent finding in animal models (Arnsten et al., 1994, 2017; Murphy et al., 1996b; Cai and Arnsten, 1997; Zahrt et al., 1997; Vijayraghavan et al., 2007; Wang et al., 2019; Yang et al., 2021). The recent and ongoing clinical testing of several D_1_ agonists of a novel chemotype (Gray et al., 2018; Sohur et al., 2018; Balice-Gordon et al., 2020; Huang et al., 2020) suggests D_1_ agonists can be used safely and for long periods. On the other hand, one of these later compounds (the D_1_ agonist PF-06412562) failed to improve cognition and motivation (Balice-Gordon et al., 2020). This compound (and several other ones) differs, however, from earlier experimental compounds in having low intrinsic activity at cAMP signaling and no intrinsic activity at β-arrestin recruitment. Earlier studies with compounds of high intrinsic activity did suggest beneficial effects of D_1_ agonists in most (Mu et al., 2007; Rosell et al., 2015; Huang et al., 2020), but not all studies (Girgis et al., 2016). These data underscore how pharmacological properties, from pharmacokinetics to signaling (Mailman and Murthy, 2010; Boyd and Mailman, 2012; Arnsten et al., 2017), must be considered above and beyond receptor selectivity. Not only does the current study highlight the crucial influence caused by the functional selectivity of drugs, it also may affect interpretation of an ongoing trial utilizing PF-06412562 (Krystal, 2019) and, as importantly, underscore the importance of detailed physiological mechanisms of D_1_ ligands.

## Data Availability Statement

The raw data supporting the conclusion of this article will be made available by the authors, without undue reservation.

## Ethics Statement

The animal study was reviewed and approved by the Institutional Animal Care and Use Committee (IACUC) Milton S Hershey Medical Center Penn State College of Medicine. All animal care and surgical procedures were in accordance with the National Institutes of Health Guide for the Care and Use of Laboratory Animals and Penn State Hershey Animal Resources Program.

## Author Contributions

YY and RM contributed to conception and design of the study. YY and SK conducted the experiments. YY performed the data analysis and wrote the first draft of the manuscript. All authors contributed to manuscript revision, read, and approved the submitted version.

## Conflict of Interest

RM has interests in issued and pending patents related to dopamine D1 receptor mechanisms that constitute a conflict of interest for which there is University oversight. The remaining authors declare that the research was conducted in the absence of any commercial or financial relationships that could be construed as a potential conflict of interest.

## Publisher’s Note

All claims expressed in this article are solely those of the authors and do not necessarily represent those of their affiliated organizations, or those of the publisher, the editors and the reviewers. Any product that may be evaluated in this article, or claim that may be made by its manufacturer, is not guaranteed or endorsed by the publisher.
